# Modeling the Dispersibility of Single Walled Carbon Nanotubes in Organic Solvents by Quantitative Structure-Activity Relationship Approach

**DOI:** 10.3390/nano5020778

**Published:** 2015-05-12

**Authors:** Hayriye Yilmaz, Bakhtiyor Rasulev, Jerzy Leszczynski

**Affiliations:** 1 Department of Biomedical Devices and Technology, Kayseri Vocational School, Erciyes University, Kayseri 38039, Turkey; 2 Interdisciplinary Center for Nanotoxicity, Department of Chemistry and Biochemistry, Jackson State University, Jackson, MS 39217, USA; E-Mails: rasulev@icnanotox.org (B.R.); jerzy@icnanotox.org (J.L.); 3 Center for Computationally Assisted Science and Technology (CCAST), North Dakota State University, Fargo, ND 58108, USA

**Keywords:** dispersibility, organic solvent, single walled carbon nanotubes (SWNTs), QSAR

## Abstract

The knowledge of physico-chemical properties of carbon nanotubes, including behavior in organic solvents is very important for design, manufacturing and utilizing of their counterparts with improved properties. In the present study a quantitative structure-activity/property relationship (QSAR/QSPR) approach was applied to predict the dispersibility of single walled carbon nanotubes (SWNTs) in various organic solvents. A number of additive descriptors and quantum-chemical descriptors were calculated and utilized to build QSAR models. The best predictability is shown by a 4-variable model. The model showed statistically good results (*R*^2^_training_ = 0.797, *Q*^2^ = 0.665, *R*^2^_test_ = 0.807), with high internal and external correlation coefficients. Presence of the X0Av descriptor and its negative term suggest that small size solvents have better SWCNTs solubility. Mass weighted descriptor ATS6m also indicates that heavier solvents (and small in size) most probably are better solvents for SWCNTs. The presence of the Dipole *Z* descriptor indicates that higher polarizability of the solvent molecule increases the solubility. The developed model and contributed descriptors can help to understand the mechanism of the dispersion process and predictorganic solvents that improve the dispersibility of SWNTs.

## 1. Introduction

With the rapid development of nanoscience and nanotechnology, carbon nanotubes (CNTs) have attracted a great deal of attention due to their unique and versatile properties since single-walled carbon nanotubes (SWCNTs) were discovered by Iijima in 1993 [1]. SWCNTs areknown for exhibiting unique mechanical, electrical, and thermal properties that are useful for a widerange of applications in materials. However, SWCNTs represent quite unusual systems and possess extremely low solubility or poor dispersibility in water and many known organic solvents [2]. Because of high polarizability, hydrophobic surface, and substantial van der Waals interactions, CNTs are able to aggregate, with each other, as well as with other chemical and biological systems to give mixture aggregates, especially in water [3,4]. The treating of CNTs by various active chemicals can change the surface properties and therefore the ability to aggregate. Some active chemicals can destruct the CNTs. This can be achieved by oxidizing CNTs by strong acids, such as refluxing in a mixture of sulfuric acid and nitric acid [5,6], “piranha” solution (sulfuric acid-hydrogen peroxide) [7], boiling in nitric acid [8], or treating with oxidative gases, such as ozone [4,9]. However, treatment under such harsh conditions clearly deviates from green chemistry and results in the opening of the tube tips [5], shortening of the tubes [7], and fragmentation of the sidewalls [8]. Therefore, the stability of CNTs may decrease, along with other important properties.

In recent years there have been several studies describing the preparation of stable suspensions of SWCNTs in a range of known solvents [9,10,11,12,13,14,15,16]. To get detailed structural information and dispersion values of SWCNTs in organic solvents, Hildebrand or Hansen solubility parameters have received close attention by researchers [17,18,19,20]. The main purpose of these studies was to improve the dispersion of nanotubes and to understand the dispersion process.

Quantitative structure activity relationships (QSARs) are often used to predict various physicochemical and biological properties of chemicals. Considering the difficulties in obtaining experimental data in CNTs research, theoretical approaches including Quantitative Structure-Property Relationships (QSPR) can provide useful information regarding predicted dispersibility values of carbon nanotubes directly from the structure. Discovering and developing effective new organic solvents for SWCNTs and C_60_ solubility have attracted many researchers’attention [21,22,23,24,25,26]. The first attempt to explain SWCNT dispersibility with the application of QSAR approach was carried out by Rofouei *et al.* [27], and the second study was made by Salahinejad *et al*. [28]. Our study is focused on developing an improved QSPR model that is able to predict the dispersibility of SWCNTs in various organic solvents.

## 2. Materials and Methods

### 2.1. Data Set

**Figure 1:**
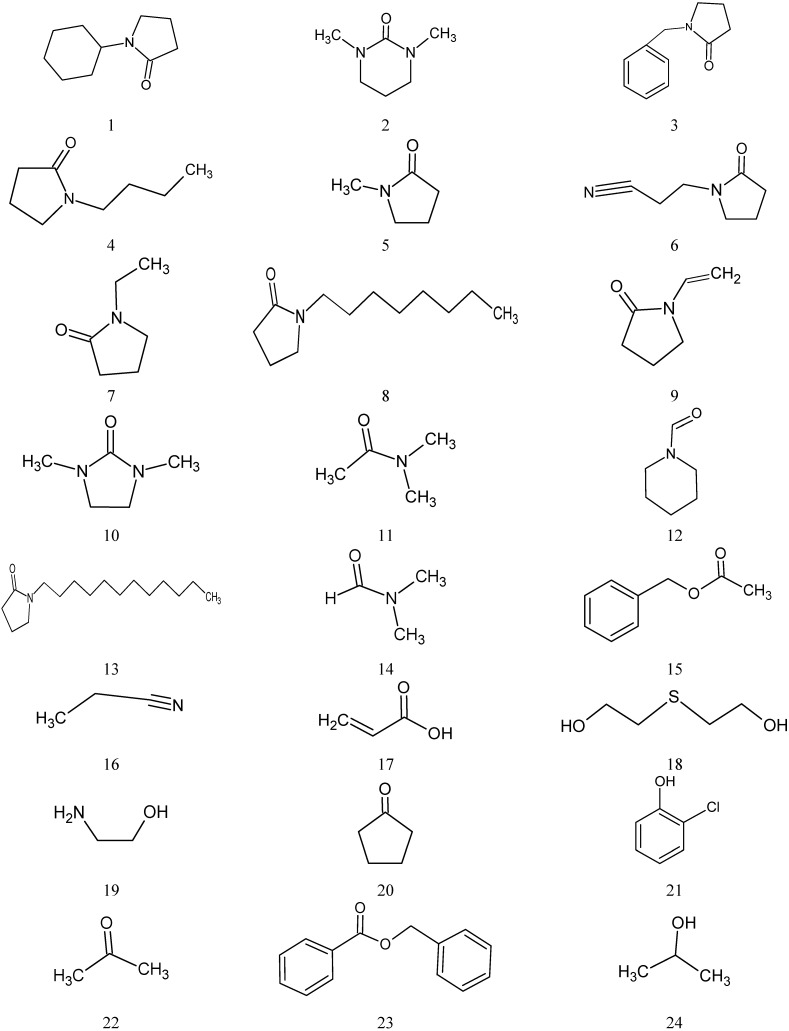

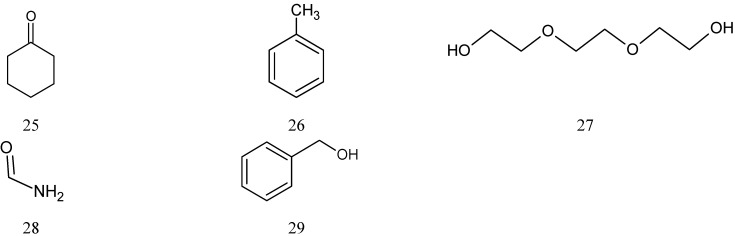
The structures of 29 organic solvents for single-walled carbon nanotube (SWCNT) derivatives.

The QSAR modeling was applied for a set of single-walled carbon nanotubes in a pool of 29 different solvents (molecular structures are represented in Figure 1) which were selected from Bergin *et al*. [29]. The set consist of 29 organic solvents, which are randomly divided into training (22 compounds) and test (7 compounds) sets. The splitting to training and test sets was balanced across the variables. The dispersibilities of studied compounds were expressed in terms of the C (mg/mL) for SWCNTs (Table 1). All original concentrations data were converted to molar LogC(exp) variables.

**Table 1:** List of 29 organic solvents used in the study, including corresponding names, experimental and calculated dispersibility of single-walled carbon nanotubes (SWCNTs) in organic solvents.

No	Name	C (mg/mL)	LogC(exp) **	LogC(cal)
1	*N*-Cyclohexyl-pyrrolidinone	3.5	0.544	0.317
2 *	1,3-Dimethyltetrahydro-2(1H)-pyrimidinone	0.65	−0.187	−0.617
3	1-Butylpyrrolidin-2-one	0.279	−0.554	−0.480
4	1-Benzylpyrrolidin-2-one	0.18	−0.745	−0.566
5 *	1-Methylpyrrolidin-2-one	0.116	−0.935	−0.972
6	3-(2-Oxo-1-pyrrolidinyl)propanenitrile	0.115	−0.939	−0.510
7	*N*-Ethyl-pyrrolidinone	0.101	−0.996	−1.200
8	*N*-Octyl-pyrrolidone	0.092	−1.036	−1.241
9	*N*-Vinyl-pyrrolidinone	0.084	−1.076	−1.459
10	Dimethyl-imidazolidinone	0.083	−1.080	−1.111
11	Dimethylacetamide	0.041	−1.387	−1.534
12 *	*N*-Formyl-piperidine	0.039	−1.409	−1.126
13	*N*-Dodecyl-pyrrolidone	0.03	−1.553	−1.157
14	Dimethylformamide	0.023	−1.638	−2.374
15	Benzyl acetate	0.0192	−1.717	−2.040
16	Propionitrile	0.015	−1.824	−1.850
17	Acrylic acid	0.0138	−1.860	−2.345
18	2,2'-thiodiethanol	0.0136	−1.866	−2.100
19 *	Ethanolamine	0.0133	−1.876	−1.424
20 *	Cyclopentanone	0.0129	−1.889	−1.757
21 *	Chlorophenol	0.012	−1.921	−2.064
22	Acetone	0.011	−1.959	−1.530
23	Benzyl benzoate	0.0109	−1.963	−2.160
24	Isopropyl alcohol	0.0105	−1.979	−2.038
25 *	Cyclohexanone	0.0068	−2.168	−2.330
26	Toluene	0.005	−2.301	−2.056
27	Triethyleneglycol	0.0037	−2.432	−2.647
28	Formamide	3.00 ×10^−4^	−3.523	−2.374
29	Benzyl alcohol	2.79×10^−4^	−3.554	−3.007

Notes: * Test Set; ** Experimental data is taken from [29].

### 2.2. Quantum Chemical Calculations

The initial structures of investigated organic solvents for SWCNTs were built using HyperChem 7.5 package [30]. After that step, the structures of compounds were firstly pre-optimized with the Molecular Mechanics Force Field (MM+) procedure included in the HyperChem. The semiempirical quantum chemical descriptors (including total energy, binding energy, electronic energy, nuclear energies, heats of formation, total dipole moment, *X*, *Y*, and *Z* components of dipole moment, E_HOMO_, E_LUMO_, surface area, volume, hydration energy, refractivity, LogP, polarizability, mass) were calculated by the RM1 method implemented in HyperChem. An initial set of 258 DRAGON software generated [31] theoretical descriptors was selected from the entire set of generated descriptors and used to describe the chemical diversity of the compounds. The software provides about 4000 various descriptors corresponding to 0D-, 1D-, 2D-, and 3D- descriptor modules. The outlined modules are comprised of 20 different classes of descriptors, namely, the constitutional, the topological, the walk and path counts, the connectivity indices, the information indices, the 2D autocorrelations, the edge adjacency indices, Burden eigenvalues, the topological charge indices, the eigenvalue based indices, the randic molecular profiles, the geometrical descriptors, the RDF descriptors, the 3D-MoRSE descriptors, the WHIM descriptors, the GETAWAY descriptors, the functional groups, the atom-centered fragments, the charge descriptors, and the molecular properties descriptors [32,33]. In addition, the density functional theory (DFT) with the hybrid meta exchange-correlation functional M06-2X/6-311G(d,p) [34] calculations were applied to obtain another set of quantum-chemically generated physico-chemical parameters of studied SWCNTs solvents—including dipole moments (total dipole moment, *X*, *Y*, and *Z* components); orbital energies, E_HOMO_, E_LUMO_ and heats of formation. All DFT calculations were performed using the Gaussian 09 software [35].

### 2.3. QSAR Modeling and Statistical Analysis

The correlation between biological activity and structural properties was obtained by using the variable selection Genetic Algorithm (GA) and Multiple Linear Analysis (MLRA) methods. Preliminary models selection was performed by means of the GA-MLRA [36,37,38] technique as implemented in the BuildQSAR [39] program. Genetic Algorithms have been applied in recent studies as a powerful tool to address many problems in QSAR studies [36,37,38]. This method based on the mechanism of evaluation of species, in which the higher descriptor weights are the more preserved in the mathematic model, while the lower weight is eliminated. In this form, the best model which represents the experimental biological activity isobtained [36,37,38,40,41]. We selected the resulting model in the range of 1–5 variables per model by limiting the GA variable selection algorithm. The MLR technique was used to develop QSAR models since it is transparent, easy interpretable, and ideal to obtain reproducible results. Several QSAR models developed were followed by statistical analysis with evaluation by squared correlation coefficient *R*^2^, standard error *s*, Fisher coefficient *F*, and non-collinearity of descriptors in the model. A final set of QSARs was tested by applying the “leave-one-out” technique (the process of removing a molecule from the set, then creating and validating the model against the individual molecules, which was performed for the entire training set). The mean was taken of all the *Q*^2^ based on the predictive error sum of squares (PRESS).

The selection of robust and well predictive QSAR models on the basis of only *R*^2^, *Q*^2^ and *R*^2^_pred_ might mislead the search for the ideal predictive model, so additional statistical analysis was done on the basis of a few other parameters, such as Average *r_m_*^2^, Delta *r_m_*^2^. For an acceptable QSAR model, the value of “Average *r_m_*^2^” should be >0.5 and “Delta *r_m_*^2^” should be <0.2 [42,43].

## 3. Results and Discussion

Our study was focused on developing a valid model that is able to predict the dispersibility of SWCNTs in various organic solvents. For this purpose we utilized a QSAR approach. Dragon software-generated additive descriptors, as well as semi-empirical and quantum mechanical descriptors were calculated and a total of 280 descriptors were used to build a QSAR model.

The correlation matrix for the most populated 2D-3D descriptors and LogC(cal) used in the present study is shown in Table 2. The sign of the correlation tells us whether the two variables are positively (more *X* means more *Y*) or negatively (more *X* means less *Y*) related. LogC(cal) and SRW09 had a good positive correlation (*r*=0.706) and strongly associated with dispersibility. In addition, LogC(cal), was found to be correlated to the Ram descriptor with *r* = 0.530 and to Dipole *Z* with *r* = 0.377, respectively. However, the correlations for any of these two descriptors considered as a single descriptor in the model were not sufficient to be considered significant in predicting dispersibility.

**Table 2:** Correlation matrix for physicochemical, 2D-3D descriptors and LogC(cal) used in the study.

Descriptor	SRW09	Dipole *Z*	piPC05	Ram	X0Av	ATS6m	LogC(cal)
SRW09	1						
Dipole Z	−0.004	1					
piPC05	−0.002	0.009	1				
Ram	0.567	0.068	0.631	1			
X0Av	0.053	−0.112	−0.275	0.075	1		
ATS6m	0.427	0.118	0.532	0.545	0.042	1	
LogC(cal)	0.706	0.377	−0.037	0.530	0.159	0.276	1

**Figure 2:**
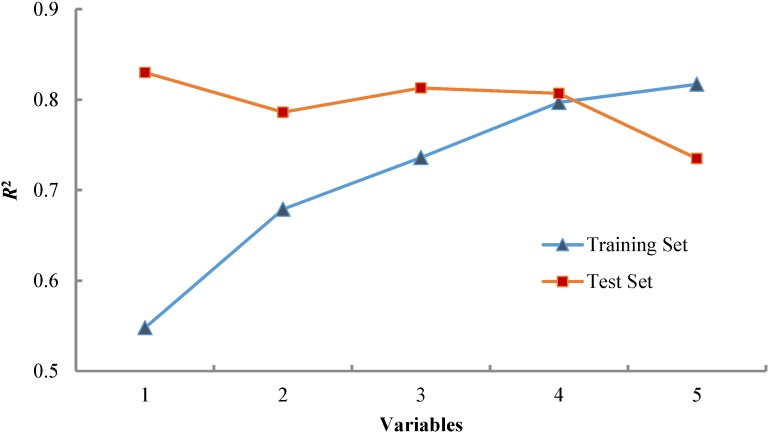
Comparison of the regression coefficients (*R*^2^) for the training and test set.

In Table 3 the performances for all developed models with 1–5 variables, for the training and test sets are listed. The 4-variable GA-MLRA based model showed the best predictive ability (*R*^2^_training_ = 0.797, *Q*^2^ = 0.665, *R*^2^_test_ = 0.807), with high internal and correlation coefficients. It is clearly noticeable that *R*^2^ values in the case of the training set follow increasing order with increase of the number of variables: 1-variable model < 2-variable model < 3-variable model < 4-variable model and for the test set follow increasing order: 2-variable model < 4-variable model < 3-variable model < 1-variable model. In the results, according to Figure 2 the 4-variable model was chosen as the most predictive and robust model.

**Table 3:** Descriptor names and statistical values for the developed models (statistics are shown for split sets into training (22 compounds) and test (7)).

No.	Descriptors	Training Set						Test Set			
		*N*	*R*^2^	*Q*^2^	*F*	SDEP	Spress	*N*	*R*^2^	*R*_m(avr)_^2^	Δ*R*^2^
**1**	SRW09	22	0.548	0.453	24.249	0.674	0.690	7	0.830	0.773	0.112
**2**	SRW09, Dipole *Z*	22	0.679	0.573	20.080	0.595	0.626	7	0.786	0.717	0.137
**3**	Ram,piPC05, Dipole *Z*	22	0.736	0.601	16.687	0.575	0.621	7	0.813	0.751	0.122
**4**	ATS6m,SRW09, X0Av, Dipole *Z*	22	0.797	0.666	16.722	0.527	0.585	7	0.807	0.744	0.125
**5**	X3A,ATS6m, SRW09, X0Av, Dipole *Z*	22	0.817	0.632	14.366	0.552	0.633	7	0.736	0.702	0.162

Table 4 represents the descriptor values selected using GA-MLR variable selection for dispersibility of SWCNTs in organic solvents. The one, two, and four variable models include SRW09 (9th order self-returning walk count). Organic solvents like 1, 3–10, 13, and 20 which have 5-membered rings, show a high value of SRW09, with a positive impact on the SWCNTs dispersibility.

The 1-variable model is represented by the following Equation (1):

Log(C)_max_ = + 0.0020 (± 0.0008) **SRW09** − 2.1522 (± 0.3620) (1)

where *n* = 22, *R*^2^ = 0.548, *s* = 0.627, *F* = 24.249, *p* = 0.0001, *Q*^2^ = 0.453, SPress = 0.690, SDEP = 0.674.

**Table 4:** The descriptor values for organic solvents.

No.	SRW09	Dipole *Z*	piPC05	Ram	X0Av	ATS6m
1	702	2.225	30	3	0.632	6.731
2	0	−0.166	10	1	0.576	0.000
3	684	1.312	78	3	0.582	7.343
4	684	0.459	14	2	0.660	6.580
5	504	0.515	4	1	0.588	0.000
6	684	1.110	18	2	0.584	6.472
7	684	−1.214	8	2	0.648	2.583
8	684	−0.743	18	2	0.673	7.406
9	684	−1.345	10	2	0.595	2.215
10	504	−0.006	4	1	0.554	0.000
11	0	0.000	0	2	0.726	0.000
12	0	2.665	12	1	0.621	3.566
13	684	−0.590	22	2	0.681	7.797
14	0	0.000	0	0	0.521	0.000
15	0	1.192	63	2	0.583	6.898
16	0	0.000	0	0	0.649	0.000
17	0	0.000	0	1	0.528	0.000
18	0	0.001	2	0	0.707	5.869
19	0	0.887	0	0	0.610	0.000
20	504	0.454	4	1	0.623	0.000
21	0	0.000	52	2	0.611	0.703
22	0	0.000	0	1	0.727	0.000
23	0	−0.763	132	3	0.550	7.631
24	0	−1.142	0	1	0.756	0.000
25	0	1.892	10	1	0.635	0.000
26	0	0.000	44	1	0.627	1.401
27	0	0.000	5	0	0.595	6.923
28	0	0.000	0	0	0.521	0.000
29	0	−0.973	50	1	0.568	3.508

**Figure 3:**
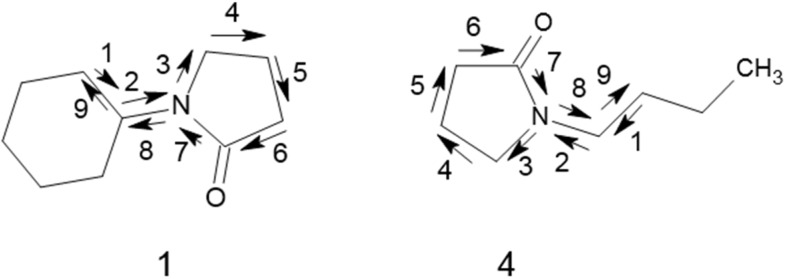
Self-returning walk of the 9th order of molecules 1 and 4.

The first descriptor given in the Equation (1) above is SRW09 (from the MWC class). It is among the 2D-descriptors representing self-returning walk counts of different lengths. The SRW count of any even order indicates the length and shape (branching) of the entire molecular graph. It increases when the number of atoms increases, when a molecule becomes more branched or contains even-membered rings. But on the other hand, the SRW count of the odd order represents only local surrounding of odd-membered rings [44,45]. To clarify the above, the self-returning walk of the 9th order of the molecules 1 and 4 structures is given in Figure 3.

SRW09 is the self-returning walk count of the 9th order and represents the surroundings of odd membered rings (five-membered in our case). It provides valuable insight into the relationship between structure and dispersibility action.

The 2-variable model is represented by following Equation (2):

Log(C)_max_ = + 0.0019 (± 0.0007) **SRW09** + 0.3809 (± 0.2865) **Dipole *Z*** − 2.1228 (± 0.3149) (2)

where *n* = 22, *R*^2^ = 0.679, *s* = 0.543, *F* = 20.080, *p* < 0.0001, *Q*^2^ = 0.573, SPRESS = 0.626, SDEP = 0.595.

The second descriptor in Equation (2) is a dipole moment descriptor, which is a 3D electronic descriptor that indicates the strength and orientation behavior of a molecule in an electrostatic field. Both the magnitude and the components (*X*, *Y*, *Z*) of the dipole moment are calculated. The descriptor is estimated by utilizing partial atomic charges and atomic coordinates. The presence and sign of the dipole moment contribution indicates that the higher polarity of the attached fragment the higher will be the overall dispersibility value of the SWCNT derivative.

The 3-variable model is represented by following Equation (3):

Log(C)_max_ = +0.8389 (± 0.2960) **Ram**− 0.0209 (± 0.0122) **piPC05** + 0.3855 (± 0.2920) **Dipole *Z*** − 2.3590 (± 0.4189) (3)

where *n* = 22, *R*^2^ = 0.736, *s* = 0.506, *F* = 16.687, *p* < 0.0001, *Q*^2^ = 0.601, SPress = 0.621, SDEP = 0.575.

Here one can see the presence of the Ram descriptor, which positively contributes to the dispersibility. The topological descriptor Ram addresses the branching in the molecule [46]. Its regression coefficient suggests in favor of more branched molecular structures for increased activity. Another descriptor represents the molecular multiple path count of order 05 (piPC05). This descriptor reflects the length of the molecule. In Equation (3) piPC05 contributes negatively to the dispersibility.

The 4-variable model is represented by the following Equation (4):

Log(C)_max_ = +0.0022 (± 0.0008) **SRW09** + 4.0933 (± 3.1813) **ATS6m** − 0.0832 (± 0.0857) **X0Av** + 0.5488 (± 0.2716) **Dipole *Z*** − 4.5062 (± 1.9741) (4)

where *n* = 22, *R*^2^ = 0.797, *s* = 0.456, *F* = 16.722, *p* < 0.0001, *Q*^2^ = 0.666, SPRESS = 0.585, SDEP = 0.527.

In the model (4) one can see the presence of other descriptors, ATS6m and X0Av. Walk and path counts class descriptors, SRW09, the 2D-AUTO class descriptors, the atomic mass weighted terms ATS6m show the higher weights with positive influences. In this model, the atomic mass weighted term (ATS6m) showed the highest contribution. The activity exhibits negative linear relationship with the connectivity index descriptor (X0Av), which is an average valence connectivity index chi-0.

Analysis of these influential molecular descriptors can lead to the revealing of the mechanism of the dispersion process and thus the model is able to predictnew organic solvents that improve the dispersibility of SWCNTs. For example, X0Av descriptor and its negative term suggest that small size solvents have better influence on the solubility of SWCNTs. Also, mass weighted descriptor ATS6m indicates that a heavier solvent (at the same time having a small size) is most probably a better solvent for SWCNTs. The presence of the Dipole *Z* descriptor indicates that higher polarizability of the solvent molecule increases the solubility.

**Figure 4:**
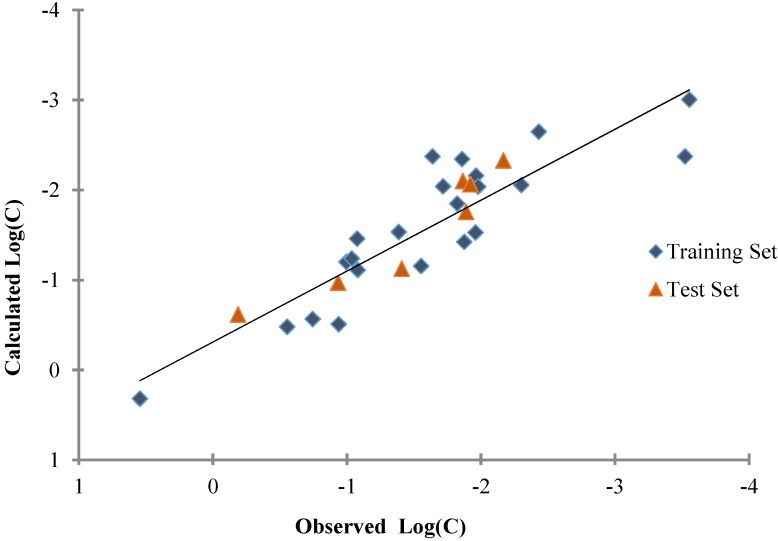
A plot of observed and predicted LogC_max_ values for the entire set (29 compounds) calculated by the 4-variable model.

The dependence of the number of variables in the models, for training and test sets, on the *R*^2^ values is displayed in Figure 2. The correlation graph of the best QSAR model (Equation (4)) is shown in Figure 4. The GA-MLRA based QSAR model with four variables showed better results than other models (*R*^2^_training_ = 0.797, *Q*^2^ = 0.666, *R*^2^_test_ = 0.807), with high internal and external correlation coefficients. A good agreement between the predictions and the experimental values confirmed the reliability of the QSAR model.

## 4. Conclusions

In the present work we investigated the influence of the characteristics of a series of organic solvents on the dispersibility of SWCNTs. For this purpose both the additive descriptors (DRAGON-software based) and quantum-chemical descriptors were generated, a total of 280 descriptors.

The 4-variable model retains also a good ratio of the number of descriptors and their predictive ability. TheGA-MLRA based model showed good results (*R*^2^_training_ = 0.797, *Q*^2^ = 0.665, *R*^2^_test_ = 0.807), with high internal and external correlation coefficients. The model (4) developed here showed the highest performance with the presence of the following four descriptors, SRW09, ATS6m, Dipole Z, and X0Av. In this model the atomic mass weighted term (ATS6m) showed the highest contribution. The other significant descriptors are graph elements weighted SRW09 (as walk and path counts) and Dipole-*Z* (as a 3D descriptor). Descriptors SRW09 and Dipole *Z* exhibit a positive influence on the dispersibility. A molecular multiple path count of the order 05 (piPC05) and an average valence connectivity index of the order 0 (X0Av) showed negative influence on the considered activity. The X0Av descriptor and its negative term suggest that small size solvents have better influence on the solubility of SWCNTs. Also, the mass weighted descriptor ATS6m indicates that a heavier solvent (at the same time having a small size) most probably is the better solvent for SWCNTs. The presence of the Dipole *Z* descriptor indicates that a higher polarizability of the solvent molecule increases the solubility. Analysis of these influential molecular descriptors can lead to details of the mechanism of the dispersion process and thus enable predictions of new organic solvents to improve the dispersibility of SWCNTs.
